# Deletion 16p13.11 uncovers *NDE1* mutations on the non-deleted homolog and extends the spectrum of severe microcephaly to include fetal brain disruption

**DOI:** 10.1002/ajmg.a.35969

**Published:** 2013-05-23

**Authors:** Alex R Paciorkowski, Kim Keppler-Noreuil, Luther Robinson, Christopher Sullivan, Samin Sajan, Susan L Christian, Polina Bukshpun, Stacy B Gabriel, Joseph G Gleeson, Elliott H Sherr, William B Dobyns

**Affiliations:** 1 Departments of Neurology, Pediatrics, and Biomedical Genetics, Center for Neural Development and Disease, University of Rochester Medical CenterRochester, New York; 2 Division of Medical Genetics, Department of Pediatrics, The University of Iowa Children’s HospitalIowa City, Iowa; 3 Division of Genetics, SUNY at Buffalo School of Medicine and Biomedical SciencesBuffalo, New York; 4 Center for Integrative Brain Research, Seattle Children’s Research InstituteSeattle, Washington; 5 Department of Neurology, University of California, San FranciscoSan Francisco, California; 6 The Broad Institute of MIT and HarvardCambridge, Massachusetts; 7 Howard Hughes Medical Institute, Institute for Genomic Medicine, Rady Children’s Hospital, University of California, San DiegoSan Diego, California; 8 Departments of Pediatrics and Neurology, University of WashingtonSeattle, Washington

**Keywords:** deletion 16p13.11, NDE1, whole exome sequencing, fetal brain disruption, microcephaly, agenesis of the corpus callosum

## Abstract

Deletions of 16p13.11 have been associated with a variety of phenotypes, and have also been found in normal individuals. We report on two unrelated patients with severe microcephaly, agenesis of the corpus callosum, scalp rugae, and a fetal brain disruption (FBD)-like phenotype with inherited deletions of 16p13.11. The first patient was subsequently found on whole exome sequencing to have a nonsense mutation (p.R44X) in *NDE1* on the non-deleted chromosome 16 homolog. We then undertook copy number studies of 16p13.11 and sequencing of *NDE1* in nine additional patients with a similar severe microcephaly, agenesis of the corpus callosum, and FBD-like phenotype. The second patient was found to have an inherited deletion of the entire *NDE1* gene combined with a frameshift mutation (c.1020-1021het_delGA) in the non-deleted *NDE1*. These observations broaden the phenotype seen in *NDE1*-related microcephaly to include FBD. These data also represent the second described syndrome, after Bernard-Soulier syndrome, where an autosomal recessive condition combines an inherited segmental duplication mediated deletion with a mutation in a gene within the non-deleted homolog. Finally, we performed informatics analysis of the 16p13.11 gene content, and found that there are many genes within the region with evidence for role(s) in brain development. Sequencing of other candidate genes in this region in patients with deletion 16p13.11 and more severe neurophenotypes may be warranted.

## INTRODUCTION

Chromosome 16 is rich in intrachromosomal segmental duplications Martin et al., 2004 that recombine to cause several recurrent copy number variants and associated clinical syndromes. Among the more common of these are deletions and duplications of 16p13.11 (chr16:14.7–16.3 Mb) that contain a core set of genes including *NDE1* Ullmann et al., 2007; Hannes et al., 2009; de Kovel et al., 2010; Heinzen et al., 2010; Mefford et al., 2010; Williams et al., 2010; Ingason et al., 2011; Ramalingam et al., 2011; Sahoo et al., 2011. Deletions of this region have been found in 1.34 per 1,000 individuals with schizophrenia and 4.9–6.0 per 1,000 individuals with various epilepsy syndromes as well as 4.4 per 10,000 controls de Kovel et al., 2010; Heinzen et al., 2010; Ingason et al., 2011. In addition to epilepsy and schizophrenia, deletion 16p13.11 has been associated with a wide spectrum of abnormalities including attention deficit hyperactivity disorder Williams et al., 2010, autism Ramalingam et al., 2011, intellectual disability, mild microcephaly, and congenital anomalies such as cleft lip, facial dysmorphism and even holoprosencephaly Ullmann et al., 2007; Hannes et al., 2009.

During the course of a copy number variant study in our cohort of patients with agenesis of the corpus callosum (ACC), we discovered the common deletion 16p13.11 in a boy with severe congenital microcephaly, ACC, collapsed skull and prominent scalp rugae that overlap with the so-called “fetal brain disruption” (FBD) sequence Russell et al., 1984; Bönnemann and Meinecke, 1990; Moore et al., 1990; Alexander et al., 1995. As his condition appeared very different from other patients with deletion 16p13.11, we performed whole exome sequencing (WES) to search for other genetic variants to explain his severe phenotype and identified a nonsense mutation in the non-deleted homolog of *NDE1*. We next performed quantitative PCR and sequencing of *NDE1* in another nine patients with severe microcephaly and an FBD-like phenotype, and found a second unrelated girl with a compound heterozygous deletion 16p13.11 and *NDE1* intragenic mutation. The phenotype in these children is intermediate between the few other individuals reported with homozygous mutations of *NDE1* Alkuraya et al., 2011; Bakircioglu et al., 2011; Guven et al., 2012.

Our observations suggest that mutations (including deletion) of *NDE1* may prove to be a relatively common cause of severe congenital microcephaly. Furthermore, the 16p13.11 deletion serves as the second paradigm for microdeletions uncovering mutations of genes on the non-deleted homolog—the other being Bernard-Soulier syndrome due to deletion 22q11.2 and mutation of *GP1BB* on the non-deleted homolog Budarf et al., 1995; Ludlow et al., 1996. Reports of single patients with novel and especially severe phenotypes should be considered provisional and likely to require more complex explanations. Finally, our informatics analysis of the gene content of the common 16p13.11 deletion provides insights into the contribution to neurologic development and function of other genes in the region.

## METHODS

### Patients

Patients were ascertained through the Molecular Genetic Studies of Developmental Disorders, and informed consent was obtained with approval of the Seattle Children’s Hospital Institutional Review Board. Retrospective clinical records and brain MRI scans were reviewed in a cohort of patients with severe microcephaly and FBD-like brain malformation. DNA was extracted from peripheral blood using the Gentra PureGene DNA isolation kit according to the manufacturer’s instructions.

### Chromosomal Microarray

LP97-141a1, with severe microcephaly and FBD, was studied using the InfiniumII HumanHap610 BeadChip high-density single nucleotide polymorphism (SNP) array (Illumina, San Diego, CA). Data were analyzed using PennCNV Wang et al., 2007 including for analysis only CNVs larger than 30 kb that contained at least 10 contiguous SNPs and at least 1 exon.

### Whole Exome Sequencing

We performed WES of peripheral blood DNA from Patient LP97-141a1 to find other genetic variants to explain his severe phenotype. We used the Nimbelgen whole exome capture kit, and sequence was generated on an Illumina GAII platform. Sequence was aligned to hg19 using BWA, and single nucleotide variants and indels were called using GATK 1.3. Mean coverage was calculated using GATK 1.3 DepthOfCoverageWalker. Annotation of variants was performed with SeattleSeq Annotation 134. Common variants were identified by filtering against the NHLBI Exome Variant Server. All tools are listed in the URLs cited.

### Sanger Sequencing

Variants found by WES were confirmed with Sanger sequencing using standard methods. We also studied by direct Sanger sequencing the coding regions of *NDE1* blood-derived DNA from products of conception (LP97-141a3, sibling of a1) from a pregnancy affected with severe microcephaly detected in utero at 14 weeks gestation, as well as eight additional patients with severe microcephaly and FBD-like phenotype. Sequence chromatograms were analyzed using the Mutation Surveyor software version 3.97 (SoftGenetics, State College, PA). Sequences were compared with normal control samples and the reference sequences for *NDE1* (NCBI reference number NM_017668).

### Quantitative PCR

The nine additional patients with severe microcephaly and FBD-like phenotype were studied with qPCR of five probes (Supplementary Table SI—see supporting information online) flanking NDE1 on 16p13.11 using standard methods Livak and Schmittgen, 2001.

### Informatics Analysis of 16p13.11 Gene Content

The gene content of published deletion 16p13.11 patients was extracted and annotated using GEDI and UCSC TableBrowser. Tissue expression and developmental stage of expression in the mouse was evaluated by query of Allen Brain Atlas, BGEM, EMAGE, and MGI. All tools are listed in URLs cited.

## RESULTS

### Clinical Phenotype

**Figure 1:**
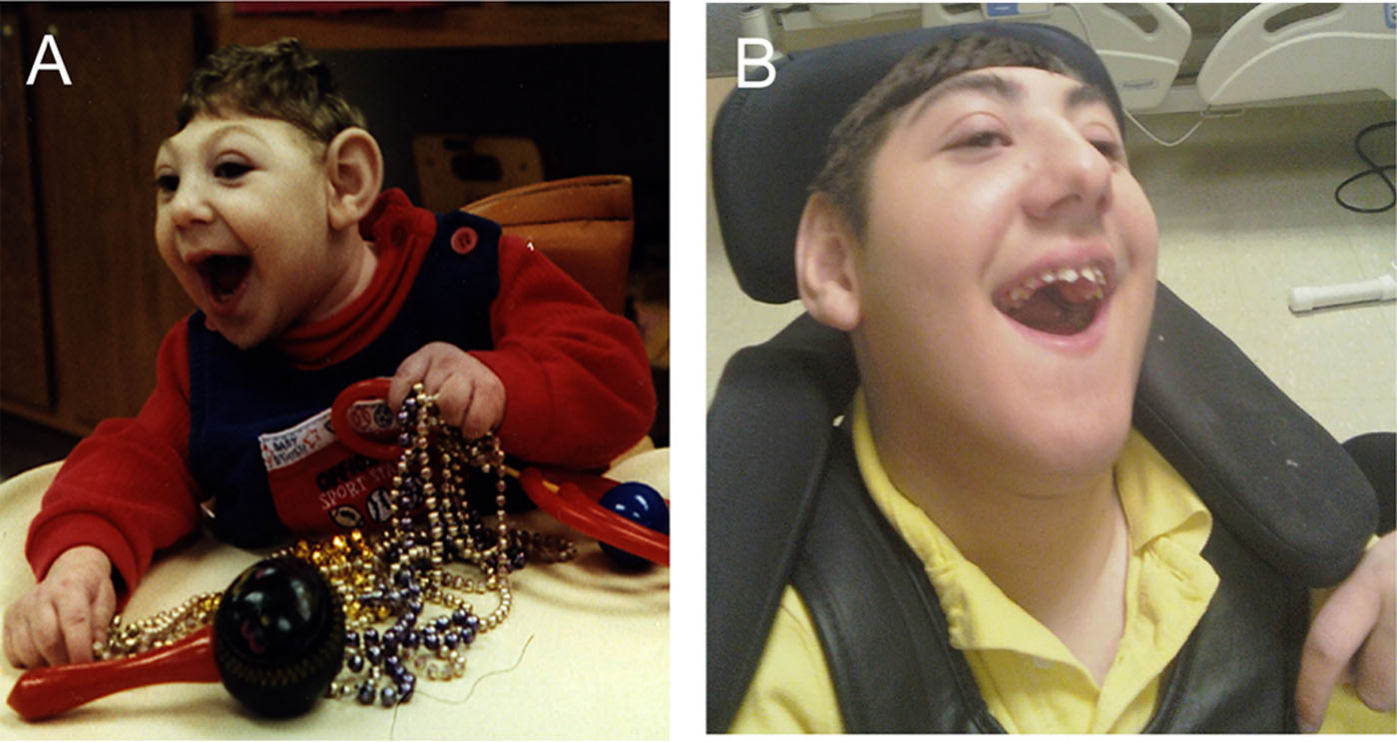
Patient LP97-141a1 at 5 months (A) and at 15 years of age (B) showing severe microcephaly, mild prognathism that developed over time, but otherwise non-dysmorphic facies.

**Figure 2:**
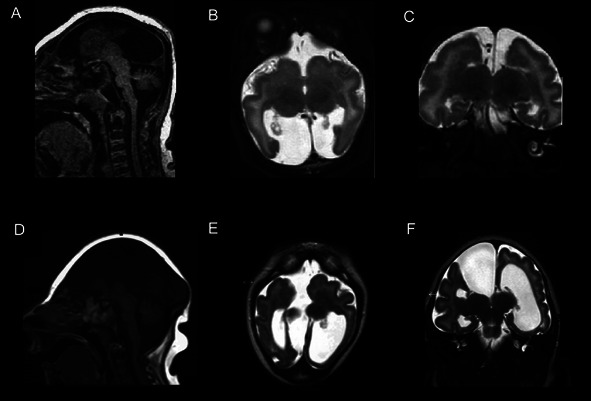
Brain MRI of Patient LP97-141a1 showing agenesis of the corpus callosum on sagittal T1 view (A). There is also increased extra-axial fluid in the posterior fossa. Axial T2 view (B) shows a very simplified gyral pattern, with underdevelopment of the posterior midline cortex at the level of the thalami. There is a polymicrogyria-like cortical dysplasia. Coronal T2 view (C) shows increased extra-axial space in the intrahemispheric fissure with communication with the left lateral ventricle. Brain MRI of Patient LR01-271 demonstrated similar agenesis of the corpus callosum, disproportionately small brainstem, and increased posterior fossa extra-axial fluid on sagittal T1 view (D). Axial T2 view at the thalami demonstrated a large interhemispheric fluid space adjacent to the right lateral ventricle (E). This fluid space is also seen on coronal T2 view (F) and appears to communicate with the right lateral ventricle.

LP97-141a1 had severe congenital microcephaly (Fig. 1), with birth occipital-frontal circumference (OFC) of −7 standard deviations (SD) below the mean. He had impaired gastric motility and required gastric bypass and placement of a gastrostomy tube at 8 months of age. He subsequently had a developmental disorder characterized by epilepsy, absence of speech and all non-verbal communication, and inappropriate laughter. Seizures appeared before the first year of life and were treated successfully with phenobarbital. Temperature instability began at age 14 years, with daily episodes of hypothermia requiring external warming. Brain MRI showed ACC and increased extra-axial space, a cortical dysplasia that appeared to be polymicrogyria-like, and a disproportionately small brainstem and cerebellum (Fig. 2A–C). The family had two subsequent pregnancies also affected with severe microcephaly and ACC, with OFC measurements at 14 weeks gestation consistent with 12 weeks gestation. A limited amount of DNA was available from the products of conception of LP97-141a3.

Patient LR01-271 was born with congenital microcephaly (birth OFC −8 SD). A single seizure was noted during infancy, but she had no further episodes. Gastroesophageal reflux disease (GERD) was treated with lansoprazole. She also subsequently had a severe neurodevelopmental phenotype that included absence of speech and all non-verbal language. She had similar hypothermic episodes that began at around 9 years of age. Brain MRI demonstrated ACC, a disproportionately small brainstem, and a large interhemispheric fluid space adjacent to the right lateral ventricle. This fluid space is also visible on coronal view and appears to communicate with the right lateral ventricle (Fig. 2D–F). She died of respiratory distress and cardiopulmonary arrest due to apparent sepsis at the age of 10 years. Both LP97-141a1 and LR01-271 were from non-consanguineous pedigrees, and the parents of both patients were phenotypically normal. The clinical findings of the patients are summarized in Table 1.

**Table 1:** Clinical Findings of Two Patients With Deletion of 16p13.11 and Mutation of *NDE1*

Patient	Birth OFC	Growth	Dysmorph	Motor	Speech	GI	Temp	EPI
LP97-141a1	−7 SD	Birth length: 25%; weight: 90%	Prominent scalp rugae at birth, large chin	Non-ambulatory	Non-verbal and no meaningful non-verbal communication	Severe dysmotility requiring gastric bypass	Hypothermia	Yes, onset during infancy, controlled on PB
		Height (15Y): <3%; weight (15Y): 25%						
LR01-271	−8 SD	Birth length: 90%; weight: 25%	Prominent scalp rugae at birth, hypocanthal folds	Non-ambulatory	Non-verbal and no meaningful non-verbal communication	GERD on medication	Hypothermia	No, single seizure during infancy
		Height (10Y): <3%; weight (10Y): <3%						

Dysmorph, dysmorphology; EPI, epilepsy; GERD, gastroesophageal reflux; GI, gastrointestinal function; OFC, occipitofrontal circumference; PB, phenobarbital; SD, standard deviations from the mean; Temp, temperature regulation; Y, years.

### Copy Number Studies

**Figure 3:**
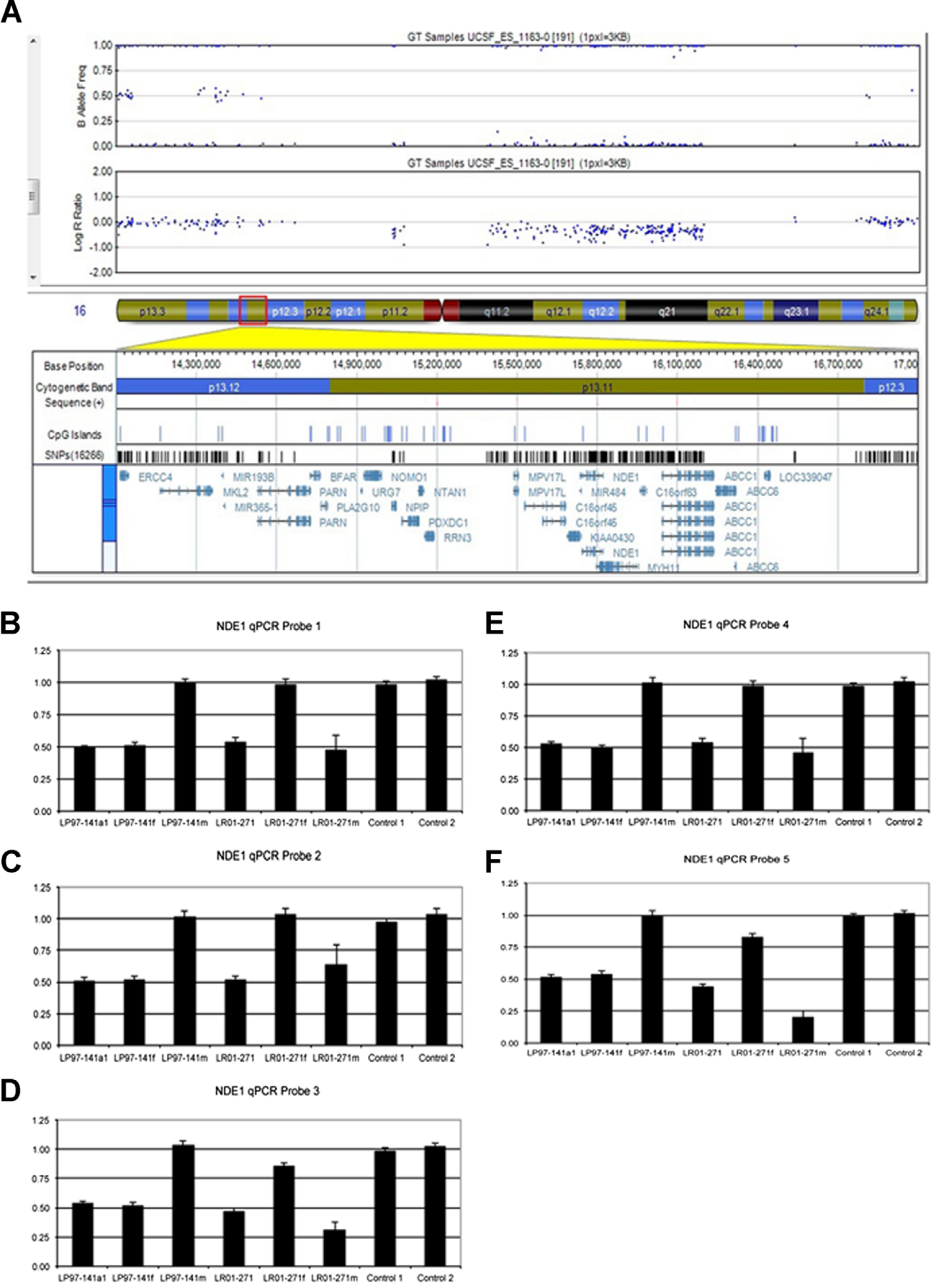
A: Deletion of 185 probes (0.83 Mb) on 16p13.11 found by chromosomal microarray in Patient LP97-141a1. B–F: Quantitative PCR of five probes flanking *NDE1* demonstrated the 16p13.11 deletion in LP97-141a1 to be inherited from the father. The deletion of *NDE1* in Patient LR01-271 was shown by qPCR to be inherited from the mother.

Patient LP97-141a1 was found by CMA to have a 0.83 Mb deletion of 185 probes on 16p13.11 (hg18 chr16:15369798-16195546x1). Quantitative PCR of proband and parental samples found this deletion to be inherited from the phenotypically normal father. The second patient, LR01-271, was found by qPCR to have a deletion of five markers flanking *NDE1* on 16p13.11. The deletion was inherited from her phenotypically normal mother. Due to limited DNA availability, copy number studies could not be performed in DNA extracted from LP97-141a3. The copy number studies are summarized in Figure 3.

### Whole Exome Sequencing

**Figure 4:**
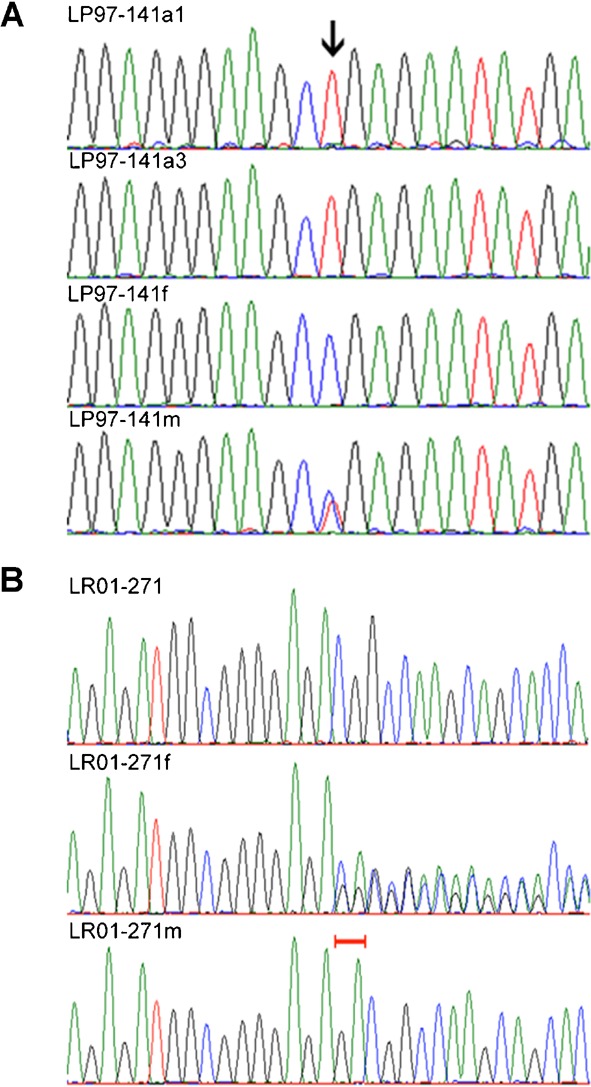
A: *NDE1* sequence chromatogram of (top to bottom) LP97-141a1, affected products of conception (LP97-141a3), father (LP97-141f), and mother (LP97-141m). Arrow shows hemizygous c.142C>T (R44X) in affected individuals, inherited from the mother. The single peak at the mutation in LP97-141a3 indicates the presence of only one allele, suggesting the deletion of the homologous copy of *NDE1*. B: Sequence chromatograms of LR01-271, father (LR01-271f), and mother (LR01-271m). The mother is hemizygous for a normal copy of *NDE1*. Red brackets show the two bases normal in the mother but heterozygously deleted in the father (c.1020-2021het_delGA). The proband inherited the mother’s total *NDE1* deletion and the father’s GA-deletion to create a compound heterozygous loss of normal *NDE1*.

In order to further understand the genetics of the severe disorder found in Patient LP97-141a1, we performed WES of blood cell-derived genomic DNA. We obtained a mean depth of coverage of 100× over the targeted exome. Our analysis identified a stop codon (chr16:15761189C/T, p.R44X) in *NDE1* that was confirmed by Sanger sequencing (Fig. 4A).

### Sanger Sequencing

We studied eight additional patients with severe microcephaly and FBD-like phenotype, and the products of conception of the affected sibling LP97-141a3. We identified the p.R44X mutation in *NDE1* in LP97-141a3. In this patient, insufficient DNA was available for copy number studies, but the Sanger chromatogram showed only one allele suggesting deletion of the other allele. The p.R44X mutation is predicted to cause protein truncation in the homodimerization domain of NDE1. In Patient LR01-271, Sanger sequencing found a 2-bp deletion (c.1020-1021het_delGA) resulting in frameshift of *NDE1*. Sequencing of parental DNA demonstrated this mutation was inherited from the father (Fig. 4B).

### Informatics Analysis of 16p13.11 Gene Content

Sixteen protein-coding genes were extracted from the 2 Mb 16p13.11 deletion region. The timing during development of gene expression, if known, and tissue(s) of expression are summarized in Supplementary Table SII—see supporting information online. Ten of the genes had evidence from at least one source of expression during brain development. Five of these genes (*ABCC1*, *NDE1*, *NOMO1*, *NTAN1*, and *PDXDC1*) had evidence from multiple sources, suggesting a role in brain development.

## DISCUSSION

We describe two unrelated patients with severe microcephaly, ACC, and FBD-like cortical malformation, who have inherited deletions of 16p13.11 combined with truncating mutations in *NDE1*. In all patients reported to date with the homozygous *NDE1* phenotype, severe congenital microcephaly was present with profound intellectual disability and early onset seizures. In the first two reports, brain imaging showed severe microcephaly, extremely simplified gyral pattern, and ACC Alkuraya et al., 2011; Bakircioglu et al., 2011, as well as normal cortical ribbon thickness and small cerebellum. The malformation was designated microcephaly with lissencephaly or “microlissencephaly” Alkuraya et al., 2011. On our review, the published images suggest a dysplastic cortex and proportionate cerebellum. We prefer to restrict use of the term “lissencephaly” to malformations in which the cerebral cortex is abnormally thick, so would describe the published images as showing microcephaly with a simplified gyral pattern and polymicrogyria-like cortical malformation. The third report shows the same severe microcephaly, growth deficiency, simplified gyral pattern and ACC, but also scalp rugae, a striking loss of brain tissue described as “microhydranencephaly” and a polymicrogyria-like cortical dysplasia Guven et al., 2012.

Our two patients also had prominent loose scalp skin with scalp rugae. Brain MRI showed changes intermediate between the prior reports, including severe microcephaly, enlarged extra-axial fluid surrounding the brain that communicated with the lateral ventricles medially, severe simplified gyral pattern with obvious polymicrogyria-like cortical malformation, and ACC. The scalp rugae suggest late fetal loss of brain volume, and combined with the dramatic increased extra-axial space, resembles an FBD-like phenotype.

FBD refers to severe congenital microcephaly with a collapsed skull and prominent scalp rugae, increased extra-axial space, and cortical clefting. This combination of features has been previously regarded as occurring secondary to intrauterine brain disruption Russell et al., 1984; Bönnemann and Meinecke, 1990; Moore et al., 1990; Alexander et al., 1995; Schram et al., 2004, and thus not genetic with a low recurrence risk. Our observations show that FBD can result from genetic causes with a high recurrence risk, although we still believe that some forms of FBD are indeed secondary to intrauterine brain disruption.

The malformations shown in the homozygous *NDE1* reports most closely resemble the pattern associated with mutations of *WDR62* Bilgüvar et al., 2010; Yu et al., 2010, while the associated growth deficiency resembles the pattern seen with severe microcephaly due to mutations of *CENPJ* Al-Dosari et al., 2010. Both *WDR62* and *CENPJ* appear to be rare causes of severe congenital microcephaly reported in few pedigrees. Our data suggest that homozygous loss of *NDE1* function may be a more common cause of autosomal recessive severe microcephaly, and may be present in outbred families. As the most common forms of severe congenital microcephaly are autosomal recessive, many clinicians do not order CMA to investigate them. We obtained an array in our proband because of the presence of ACC. We now prefer to order SNP microarrays in children with severe congenital microcephaly both to detect this microdeletion and to detect stretches of homozygosity that may represent loci harboring pathogenic mutations.

This is the second segmental duplication-mediated deletion associated with a recurrent autosomal recessive phenotype. The first is the bleeding disorder Bernard-Soulier syndrome, which is caused by homozygous or compound heterozygous mutations of the *GP1BB* gene encoding the platelet glycoprotein GPIb/IX receptor. At least four patients have been reported in which the common 22q11.2 deletion served as one of the two mutations Budarf et al., 1995; Ludlow et al., 1996; Lascone et al., 2001; Nakagawa et al., 2001.

The inherited 16p13.11 deletions in our patients are additionally interesting because of the disparate phenotype reports associated with copy number variations at this locus. Many of the patients published have had relatively mild phenotypes such as ADHD, treatment-responsive epilepsies without cognitive impairment, mild microcephaly (OFC −3 SD), and isolated dysmorphologic findings such as cleft palate. It has been difficult to then reconcile the association of the same 16p13.11 deletion with the more severe neurodevelopmental findings of autism, intellectual disability, and schizophrenia. Our data suggest that these more severe phenotypes may be indicative of second genomic events—such as mutations of genes within the non-deleted 16p13.11 homolog.

Haploinsufficiency of *NDE1* has been suggested as a possible cause for the developmental and neurologic phenotypes associated with 16p13.11 deletions de Kovel et al., 2010; Heinzen et al., 2010; Mefford et al., 2010, although all parents carrying heterozygous mutations of the gene were reported to be normal Alkuraya et al., 2011; Bakircioglu et al., 2011; Guven et al., 2012. Our informatics analysis of the gene content of the 16p13.11 region found evidence that several of the genes in the region, such as *ABCC1*, *NOMO1*, *NTAN1*, and *PDXDC1* have expression during brain development—as does *NDE1*. These may be candidate genes for second-hit mutations in patients with deletions of 16p13.11 and more severe disorders of cortical connectivity such as autism, intellectual disability, and schizophrenia. Sequencing of these candidates in patients with severe neurodevelopmental phenotypes may yield more compelling explanations for these disorders.

## URLS CITED:

Allen Brain Atlas (http://www.brain-map.org/)

BGEM (http://www.stjudebgem.org/web/mainPage/mainPage.php)

BWA (http://bio-bwa.sourceforge.net/bwa.shtml)

EMAGE (http://www.emouseatlas.org/emage/home.php)

GATK 1.3 (http://www.broadinstitute.org/gsa/wiki/index.php/Home_Page)

GEDI (http://gedi.ci.uchicago.edu/)

MGI (http://www.informatics.jax.org/)

NHLBI Exome Variant Server (http://evs.gs.washington.edu/EVS/)

SeattleSeq Annotation 134 (http://snp.gs.washington.edu/SeattleSeqAnnotation134/)

UCSC TableBrowser (http://www.genome.ucsc.edu/cgi-bin/hgTables)
