# Supplementary material for: Deletion 16p13.11 uncovers NDE1 mutations on the non-deleted homolog and extends the spectrum of severe microcephaly to include fetal brain disruption
Source: Am J Med Genet A. 2013 May 23;161(7):1523–30. doi: 10.1002/ajmg.a.35969 (PMC3689850; doi:10.1002/ajmg.a.35969)
Supplement: Supplementary file 1 [file ajmg0161-1523-sd1.doc]

Supplementary Table 1: Primers for target regions flanking *NDE1* used in quantitative PCR.

| **Primer** | **Sequence** |
| --- | --- |
| NDE1_qPCR_F1 | GCAGGTTAGTGGCACACAGA |
| NDE1_qPCR_R1 | GGCAAGTGTTAATGCCTGGT |
| NDE1_qPCR_F2 | TCCTAGAGGTGCGTTCGAGT |
| NDE1_qPCR_R2 | CAGGGGACTCAGGAAAATGA |
| NDE1_qPCR_F3 | CAGTTCCTTCTTGGCTGAGG |
| NDE1_qPCR_R3 | TCCTGCCTTCTGCATCTTTT |
| NDE1_qPCR_F4 | GCTGAAGCCTGTTCTTGGTC |
| NDE1_qPCR_R4 | AAGCTCTGGAAGAGGGGAAG |
| NDE1_qPCR_F5 | GAGTCCAAACTCGCTTCCTG |
| NDE1_qPCR_R5 | TGACCCCTTATCACCCAAAG |
