# Supplementary material for: Deletion 16p13.11 uncovers NDE1 mutations on the non-deleted homolog and extends the spectrum of severe microcephaly to include fetal brain disruption
Source: Am J Med Genet A. 2013 May 23;161(7):1523–30. doi: 10.1002/ajmg.a.35969 (PMC3689850; doi:10.1002/ajmg.a.35969)
Supplement: Supplementary file 2 [file ajmg0161-1523-sd2.doc]

Supplementary Table 2: The timing of gene expression during mouse brain development, if known, of 12 genes located within the commonly deleted region of 16p13.11. *LOC339047,* *LOC399491, NOMO3,* and *NPIP* were excluded due to lack of data in all categories.

| **Gene** | **Allen Brain Atlas** | **BGEM** | **EMAGE** | **MGI** |
| --- | --- | --- | --- | --- |
| *ABCC1* | Not expressed in any brain regions assayed at P56 | ND | Head mesenchyme at TS15 | Future brain at TS15; Brain at TS28 |
| *ABCC6* | Not expressed in any brain regions assayed at P56 | NA | ND | Throughout embryo at TS20-26 |
| *C16orf45* | ND | ND | ND | Brain, hypothalamus, diencephalon, telencephalon, brainstem at TS23 |
| *C16orf63* | ND | ND | ND | Cortical plate, cortical subventricular zone and ventricular layer at TS22 |
| *KIAA0430* | Modest expression in all brain regions assayed (except not in cerebellum) at P56 | ND | ND | Brain, cortex, striatum, ventricular layer at TS21; Brain, hypothalamus, thalamus, cortex, hippocampus, hindbrain at TS23 |
| *MPV17L* | Highest expression in isocortex, hippocampal formation, subcortical plate, striatum, and thalamus at P56 | ND | ND | ND |
| *MYH11* | Expressed in all brain regions assayed at P56 | ND | ND | Brain, cortex, striatum, hippocampus, cerebellum, midbrain at TS28 |
| *NDE1* | Expressed in all brain regions assayed at P56 | ND | Telencephalon, midbrain, diencephalon lateral wall ventricular layer at TS23 | Diencephalon, telencephalon, midbrain at TS23; Brain at TS24-28 |
| *NOMO1* | Expressed in all brain regions assayed at P56 | Ganglionic eminence, hindbrain, hippocampal formation, hypothalamus, midbrain, neocortex, at E15; amygdala, basal ganglia, brainstem, cerebellum, cortex, hippocampus, hypothalamus, midbrain, thalamus at P7 | Throughout embryo at TS23 | Diencephalon, telencephalon, hindbrain, midbrain at TS19; Hypothalamus, neocortex, hippocampus, hindbrain, cerebellum, midbrain at TS23; Brainstem, choroid plexus, hypothalamus, thalamus, basal ganglia, amygdala, cortex, hippocampus, cerebellum, midbrain at TS28 |
| *NTAN1* | Expressed in all brain regions assayed at P56 | NA | Head mesenchyme at TS23 | CNS, choroid plexus, diencephalon, pituitary, hypothalamus, thalamus, telencephalon, amygdala, basal forebrain, basal ganglia, cortex, striatum, hippocampus, pallidum, hindbrain, cerebellum, brainstem at TS22 |
| *PDXDC1* | Highest expression in isocortex, hippocampal formation, subcortical plate at P56 | Ganglionic eminence, hindbrain, hippocampal formation, hypothalamus, midbrain, neocortex at E15; Amygdala, basal ganglia, brainstem, cerebellum, cortex, hypothalamus, midbrain at P7 | ND | Diencephalon, telencephalon, hindbrain at TS19; Hypothalamus, neocortex, ganglionic eminence, hippocampus, hindbrain, cerebellum at TS23; Brainstem, choroid plexus, hypothalamus, basal ganglia, amygdala, cerebral cortex, hippocampus, cerebellum, midbrain at TS28 |
| *RRN3* | Expressed in all brain regions assayed at P56 | ND | ND | ND |

Abbreviations: CNS = central nervous system; E=embryonic day; NA = not available; ND = no data; KO = knockout; P=postnatal day; TS = Theiler stage
